# Editorial: *Klebsiella pneumoniae*: Antimicrobial resistance, virulence and therapeutic strategies

**DOI:** 10.3389/fcimb.2022.1108817

**Published:** 2022-12-23

**Authors:** Ning Dong, Ruichao Li, Yichyi Lai

**Affiliations:** ^1^ Department of Medical Microbiology, School of Biology and Basic Medical Sciences, Suzhou Medical College of Soochow University, Suzhou, China; ^2^ Suzhou Key Laboratory of Pathogen Bioscience and Anti-infective Medicine, Soochow University, Suzhou, China; ^3^ Jiangsu Co-innovation Center for Prevention and Control of Important Animal Infectious Diseases and Zoonoses, College of Veterinary Medicine, Yangzhou University, Yangzhou, China; ^4^ Institute of Comparative Medicine, Yangzhou University, Yangzhou, China; ^5^ Department of Internal Medicine, Chung Shan Medical University Hospital, Taichung, Taiwan, China; ^6^ Department of Microbiology and Immunology, School of Medicine, Chung Shan Medical University, Taichung, Taiwan, China

**Keywords:** *klebsiella pneumoniae*, antimicrobial resistance (AMR), virulence factors, pathogenesis, host-pathogen interaction, molecular epidemiology, genomic analysis

*Klebsiella pneumoniae* is a member of the ESKAPE organisms which include 6 well-known highly virulent and antimicrobial-resistant clinical pathogens (Dong et al., 2022). *K. pneumoniae* has gained the ability to acquire external genetic materials that enable it to undergo constant evolution (Russo and Marr, 2019). The pathotypes reported to be associated with infections included classical (cKP) and hypervirulent *K. pneumoniae* (hvKP), with the former known for the capacity to acquire resistance to a wide range of antibiotics and the latter exhibiting high pathogenicity and leading to high mortality in otherwise immunocompromised hosts (Paczosa and Mecsas, 2016). To date, more than 100 distinct acquired antimicrobial resistance genes have been identified in *K. pneumoniae* (Wyres and Holt, 2016). Furthermore, apart from the well-established factors that help *K. pneumoniae* escape the innate immune mechanisms of the host, new virulence factors have been discovered (Walker et al., 2019; Walker et al., 2020; Gomes et al., 2021). Continuous effort is necessary to better understand this ‘superbug’ and help design feasible approaches to eradicate or halt its further evolution. As a step towards countering the threat of *K. pneumoniae*, our Research Topic brings a collection of seven selected articles exploring its molecular epidemiology, antimicrobial resistance, and pathogenesis.

*K. pneumoniae* is a common pathogen of community-acquired pneumonia (CAP) in Asian countries (Song et al., 2008). Chen et al. revisited the burden of *K. pneumoniae* bacteremic pneumonia (KPBP) and determined the risk factors associated with 28-day mortality by analyzing data from 150 patients with KPBP in Taiwan from 2014-2020. A remarkably high 28-day mortality was observed in all patients. hvKP was more prevalent in CAP than in nosocomial pneumonia, yet carbapenem-resistant *K. pneumoniae* (CRKP) was more prevalent in nosocomial pneumonia than in CAP. Nosocomial pneumonia, Severe Organ Failure Assessment core, and lack of appropriate definitive treatment were positive predictors for 28-day mortality among patients with KPBP. The results suggested host factors, disease severity, and timely effective therapy could affect the treatment outcomes of patients with KPBP.

Information regarding hvKp infections in pediatric patients remains limited. Du et al. characterized *K. pneumoniae* strains from a children’s hospital in Shanghai during 2019-2020. They found KPC-2-producing KL47-ST11 hypervirulence genes-positive (hgKp) increased dramatically from 5.3% in 2019 to 67.6% in 2020, suggesting genetic convergence of virulence and carbapenem-resistance in *K. pneumoniae* is increasing among children. hgKp could be classified into hvKP (32.5%) and hgKp-low virulence strains (67.5%). hvKp infections in children were mostly hospital-associated and commonly involved severe pneumonia. In hvKp, diverse genetic clones were observed and K1-ST23 and K2-ST25 were the dominant clones. These findings suggested the dramatic spread of hvKP in children.

*K. pneumoniae* strains resistant to the last-resort antibiotic, ceftazidime-avibactam (CZA) have been increasingly reported recently. Bongiorno et al. characterized 16 CZA-resistant KPC-producing *K. pneumoniae* strains from Italy. The strains were from three major clones, ST101, ST307, and ST512. All strains carried *bla*_KPC_. Most strains carried *bla*_KPC-3_ (62.5%), and other strains carried other variants (*bla*_KPC-28_, *bla*_KPC-31_, *bla*_KPC-34_, and *bla*_KPC-50_). Besides, frameshift mutations on OmpK35 and OmpK36 were observed in 15/16 strains. These results suggested CZA resistance in *K. pneumoniae* arises through both the spread of epidemic clones and the horizontal dissemination of *bla*_KPC_ variants.

Gastrointestinal carriage is a major reservoir of *K. pneumoniae* infection (Gorrie et al., 2017). Migliorini et al. investigated the mechanisms associated with the transition from carriage to infection by *K. pneumoniae* isolates carrying *bla*_KPC_ by characterizing KPC-producing strains isolated within a 10-year period. They showed the presence of resistance and virulence mechanisms were not associated with progression from colonization to infection, while intestinal colonization by carbapenem-resistant Enterobacteriaceae and, more specifically, the load of gastrointestinal carriage emerged as an important determinant of infection.

ST11 is reported to be the dominant CRKP clone in China (Qi et al., 2011). TMexCD1-TOprJ1 is the first plasmid-borne RND-type tigecycline resistance determinant reported recently (Lv et al., 2020). Li et al. characterize two clinical ST11 CRKP strains co-harboring the gene cluster *tmexCD2-toprJ2* and the metallo-β-lactamase gene *bla*_NDM-1_ on the IncFIB(Mar)-like/HI1B-like group of hybrid plasmids. One of the two strains also carried *bla*_KPC-2_ on an IncN/U plasmid. Dissemination of *tmexCD-toprJ* in clinical high-risk CRKP clones may have exacerbated the antimicrobial resistance crisis.

The *Klebsiella* genus comprises a wide diversity of species apart from *K. pneumoniae* (Dong et al., 2022). Drug-resistant *K. michiganensis* strains have been increasingly reported recently. Li et al. reported the first clinical multidrug-resistant *K. michiganensis* strain co-harboring two *bla*_SIM-1_, one *mcr-9.2*, and 23 other resistance genes. They further comprehensively investigated the population structure and antibiotic-resistance genes of *K. michiganensis* by studying 275 publicly available genomes. Two major clades were identified, with the most popular sequence type ST29 being located in Clade 1, while other common STs (such as ST50, ST27, and ST43) in Clade 2. 25.5% *K. michiganensis* harbored at least one carbapenemase gene. ST27 isolates possess the most drug-resistance gene number among all the STs. The results of this study improved the understanding of *K. michiganensis*.

Infection caused by cKP represents a significant challenge due to its rising antibiotic resistance. Mackel et al. dissected the adaptive immune responses elicited by live cKp infection and investigated how these responses protected the host from reinfection by using a lung inoculation plus challenge model. They found circulating antibody responses alone were not sufficient to mediate protection against cKP, yet either of the major T cell subsets alone (γδ or αβ) was sufficient to mediate protection; also, the circulating T cell pool was not required for the protective phenotype. These findings altogether demonstrated the imperative contribution of T cells to protective immunity against cKP which would guide further inquiries into host effector responses required to control cKP infection.

The articles presented in this collection provide a valuable addition to the understanding of the notorious pathogen, *K. pneumoniae*. We hope these contributions will help investigators in their continuous scientific pursuit to tackle issues around the antimicrobial resistance and hypervirulence of this superbug. Related fields such as accurate and rapid pathogen detection and the design of optimal treatment regimens warrant further attention.

## Author contributions

All authors listed have made a substantial, direct, and intellectual contribution to the work and approved it for publication.
